# New insights into the mechanisms of anandamide-induced airway dilation placing its degradation enzyme, FAAH center stage. Commentary on: Simon A, von Einem T, Seidinger A, Matthey M, Bindila L, Wenzel D (2022) The endocannabinoid anandamide is an airway relaxant in health and disease. Nat Commun 13:6941

**DOI:** 10.1007/s00424-023-02802-2

**Published:** 2023-03-01

**Authors:** Gabriele Pfitzer

**Affiliations:** Institute of Vegetative Physiology, Medical Faculty, University of Cologne, Robert-Koch-Str. 39, 50931 Cologne, Germany

The *Cannabis sativa* plant has been used over thousands of years in traditional medicine as ailment for a number of health problems including the pulmonary system [5]. The two most abundant phytocannabinoids, Δ^9^tetrahydrocannabinol (THC) and cannabidiol (CBD) and the later discovered endocannabinoids, N-arachidonyl ethanolamine (anandamide, AEA) and 2-arachidonylglyerol (2-AG) activate the same G-protein coupled receptors, CB1 and CB2 [5]. The endocannabinoids, the cannabinoid receptors, and the biosynthetic and degradative enzymes are collectively referred to as endocannabinoid system, components of which are expressed in all organs [5] including the lung [7] affecting organ homeostasis. Cannabis may have beneficial effects in the lung as smoking marijuana exerts potent bronchodilation. However, in some asthmatic patients, it paradoxically induces bronchospasm (references in [1]). It is to be expected that a better understanding of the pulmonary endocannabinoid system likely will open tailored treatment options [10]. The limited number of preclinical studies that addressed the effect of endocannabinoids on airway smooth muscle tone yielded divergent results as to whether or not AEA relaxed airways [1, 3, 7–9, 11], but in most reports, its effects were ascribed to activation of cannabinoid receptors on axon terminals of airway nerves [1, 3, 8, 9, 11].

Understudied is the fact that degradation of anandamide by fatty acid amide hydrolase (FAAH) generates the bioactive lipid, arachidonic acid [5], the precursor of eicosanoids known to affect airway smooth muscle tone [2]. In this new contribution, Daniela Wenzel and her group [7] provide compelling evidence that relaxation of murine airways by anandamide (AEA) is not mediated by cannabinoid receptors but rather by FAAH-dependent degradation products. The evidence is based on an impressive range of sophisticated physiological as well as biochemical and immuno-histochemical approaches in mice. First, they established that AEA relaxes pharmacologically (5-HT) pre-constricted tracheal ring preparations. Then, using CB1/CB2 null mice, they established that relaxation did not depend on cannabinoid receptors but rather on FAAH, as relaxation was absent in FAAH null mice. Active FAAH is expressed in mouse airways [7]. In line with these experiments, an inhibitor of FAAH prevented AEA-induced relaxation, and a non-hydrolysable AEA analog was ineffective. It is of interest that relaxation was reduced by ~ 60% in mechanically epithelial-denuded ring preparations of the trachea, indicating a significant contribution of an epithelium-dependent mechanisms. In an elegant series of experiments using the highly sophisticated technology of lung slice preparations, the authors demonstrated that FAAH-dependent metabolites of AEA also relax small intrapulmonary airways.

The authors then went on to identify the AEA-metabolites responsible for the FAAH-dependent relaxation. Arachidonic acid, which itself potently relaxed the trachea preparations, is metabolized to eicosanoids by cyclooxygenase (COX), 5-lipoxygenase (5-LOX), and CYP450 [2]. Using small molecule inhibitors, the authors found that the COX inhibitor, indomethacin, but not inhibitors of the other enzymes attenuated AEA-induced relaxation [7]. COX generated products are the potent smooth muscle relaxants, prostacyclin (PGI) and prostaglandin E (PGE) [2]. By blocking the respective IP and EP receptors, they found that AEA-induced relaxation involves the PGE receptors, EP2 and EP4, a finding that was corroborated by the AEA-induced increase of PGE production in human airway epithelial and smooth muscle cells along with a small increase in cAMP levels in those cells [7].

These findings open interesting therapeutic avenues. Bronchospasms are treated frequently with ß-adrenoceptor agonists. Unfortunately, prolonged application of these leads to desensitization of smooth muscles to their relaxing effect. The authors showed that in contrast to β-adrenoceptor agonists, prolonged treatment with AEA does not desensitize smooth muscle neither to AEA nor to ß-adrenoceptor agonists [7].

In the next step, the authors investigated whether AEA would be effective in vivo and in a murine asthma model, the ovalbumin (OVA)-sensitized mice. Inhalation of AEA, but not of the non-hydrolysable AEA analog prevented the increase in airway resistance induced by 5-HT. Thus, FAAH-dependent metabolites of AEA mediate airway relaxation not only in vitro but also in vivo. In OVA-sensitized mice, the authors found decreased levels of AEA, 2-AG, and of several enzymes responsible for their biosynthesis [7]. They proposed that alterations of the endocannabinoid system may contribute to the hyper-contractile phenotype in these mice. At the same time, airway relaxation by exogenously applied AEA was not impaired both ex vivo and in vivo. Thus, AEA is an efficient airway relaxant not only in healthy but also in asthmatic mice. In asthma, reduction of airway resistance not only involves relaxation of smooth muscle but also amelioration of inflammation. In this context, it is of interest that FAAH-dependent AEA metabolites were beneficial in a preclinical model of pulmonary inflammation [4]. These preclinical models [4, 7] suggest that the AEA/FAAH pathway may be a promising therapeutic target. However, the results have to be taken with some care: other studies found increased basal AEA levels in asthmatic patients [12] and increased AEA-induced permeability in human epithelial cells [6] indicating that AEA may have pro-inflammatory effects under certain conditions.

This well-designed study raises several important questions. First, are the epithelium-independent and epithelium-dependent relaxation mediated by the same FAAH-dependent metabolites? Second, are local nerves involved in the effects? This said the current study is an important step in the understanding of pulmonary endocannabinoid system placing FAAH, which links the endocannabinoid with the eicosanoid system, center stage.

## Data Availability

Not applicable.
